# Factors Associated With Nonattendance for Osteoporosis Evaluation Following Fragility Fracture

**DOI:** 10.1155/joos/5602020

**Published:** 2024-11-29

**Authors:** Thany Seyok, Jamie E. Collins, Cole Hodys, Samantha J. Erikson, Samantha Perez Menendez, Brandon E. Earp, Julia F. Charles

**Affiliations:** ^1^Department of Medicine, Division of Rheumatology, Brigham and Women's Hospital, Harvard Medical School, Boston, Massachusetts, USA; ^2^Department of Orthopaedic Surgery, Brigham and Women's Hospital, Harvard Medical School, Boston, Massachusetts, USA

**Keywords:** fragility fracture, osteoporosis, patient adherence, post-fracture care

## Abstract

**Introduction:** This study assessed patient demographic factors associated with nonattendance for osteoporosis evaluation after being referred to our Bone Health Clinic (BHC), a hospital-based outpatient Fracture Liaison Service (FLS), for a fragility fracture.

**Methods:** 507 patients sustaining a fragility fracture were referred to the BHC over a 39-month period. Retrospective chart review was conducted to capture osteoporosis evaluation attendance rates and demographic factors (age, gender, race, area deprivation index, insurance type, and fracture type). A post-fracture follow-up visit with either the BHC or another provider in which osteoporosis was noted in the assessment was considered attendance for osteoporosis evaluation. Nonattendance was determined at a cutoff of one year after the fracture date.

**Results:** Of the 507 patients referred to the BHC following a fragility fracture, 177 patients attended osteoporosis evaluation with either the BHC or a primary care provider. Nonattendance was associated with older age (*p*=0.0075), having private health insurance (*p*=0.0434), and recent hip fracture (*p* < 0.0001). Attendance was associated with having government health insurance (*p*=0.0103).

**Conclusion:** Inpatient evaluation and treatment for osteoporosis should be considered in patients who are older or have sustained a hip fracture as they may have more difficulty attending post-fracture appointments.

## 1. Introduction

Osteoporosis is a growing concern worldwide, causing over 8.9 million fractures annually [1]. Since bone loss occurs silently over time, patients are often unaware they are osteoporotic until after sustaining a fragility fracture. An estimated one in three women and one in five men over the age of 50 will suffer a fragility fracture [2–5]. Furthermore, an initial fragility fracture increases the risk of subsequent fracture, particularly within the first 2 years [6, 7]. Given the significant morbidity [8, 9], mortality [10], and financial burden [11, 12] associated with fractures, an initial fragility fracture should prompt osteoporosis screening and treatment initiation, if warranted, to prevent subsequent fractures.

Fracture Liaison Services (FLSs) have emerged as a secondary fracture prevention mechanism through identifying, evaluating, and treating patients at high risk for osteoporosis, particularly those presenting with an initial fragility fracture. Numerous studies have demonstrated the effectiveness of FLS at reducing the risk of secondary fractures, post-fracture mortality, and costs [13, 14]. Despite this, there remains an osteoporosis care gap [15], with as few as 20% of fragility fracture patients being adequately evaluated and treated for osteoporosis [16–18].

Patient attendance for osteoporosis evaluation and treatment is critical for closing the osteoporosis care gap. Evaluation of FLS attendance rates and the factors contributing to attendance are understudied. A recent systematic review and meta-analysis assessing the effect of FLSs on secondary fractures and mortality reported that FLS attendance rates are highly variable, ranging from 20% to 86% [19]. However, only six of the 16 studies cited in the review had available data to adequately evaluate attendance rates. Furthermore, reasons for low attendance are rarely reported, thus creating a challenge to identifying solutions. The objective of this study was to assess patient demographic factors associated with attendance or nonattendance for osteoporosis evaluation following a fragility fracture.

## 2. Methods

### 2.1. Study Cohort

Institutional review board approval was obtained for this retrospective chart review study, conducted at an urban academic hospital. The study period was 39 months, from October 2016, initial opening of the BHC, to December 2019, right before COVID-19 lockdowns began in 2020. During this period, 542 patients were referred to our hospital-based outpatient FLS, called the BHC, following a fragility fracture (including distal radius, proximal humerus, hip, pelvis, and spine). Patients referred for nonfracture reasons (*n* = 17) were excluded from the analytic cohort. For patients with multiple referrals to the BHC within the study period (*n* = 18), only the first referral was included in the analytic cohort to maintain the assumption of independent observations. After exclusions, a total of 507 patients comprised the analytic cohort.

### 2.2. Study Outcome

Retrospective chart review was conducted to capture osteoporosis evaluation attendance and gather demographic factors (age, gender, race, area deprivation index, health insurance type, and fracture type). Area deprivation index (ADI) is a measure of neighborhood socioeconomic disadvantage at the zip code level, and scores for our cohort were obtained through the Neighborhood Atlas [20, 21], from University of Wisconsin-Madison School of Medicine and Public Health. Health insurance type was categorized into private, government (Medicaid, Medicare, or MassHealth), motor vehicle accident (MVA) and worker's compensation, or self-pay. Fracture type was determined by chart review, analyzed as a categorical variable, and categorized as hip fracture vs. any other fracture type.

The primary outcome of this study was attendance for osteoporosis evaluation within one year of the fragility fracture. Our electronic health record system allows us to review medical records from all providers within our institution. Patients were considered to have attended osteoporosis evaluation if they had a post-fracture follow-up visit with either the BHC or another provider (includes PCP, endocrinologists, and rheumatologists) in which osteoporosis was documented in the assessment section of the visit note.

### 2.3. Statistical Methods

The study population was from a convenience sample consisting of patients referred to the BHC following a fragility fracture over a 39-month period. Continuous variables were reported as means and assessed for normality before analysis. A two-tailed *t* test was used to assess differences in normally distributed, continuous demographic variables between groups. Fisher's exact test or chi-square test was used to compare nominal variables as appropriate. A modified Poisson regression model with robust error variance [22] was used to assess associations between age and hip fracture with osteoporosis evaluation attendance in unadjusted and adjusted models. *p* values < 0.05 were considered significant.

## 3. Results

Patients referred to the BHC were predominantly older, white, non-Hispanic, and English-speaking females (Table 1). Of the 507 patients in the analytic cohort, 177 (34.91%) attended osteoporosis evaluation with either the BHC or another physician within one year of fracture. The median time from fracture to osteoporosis evaluation was 84 days. Out of the 177 patients that had osteoporosis evaluation, 85 (48.02%) followed up with the FLS and 92 (51.98%) followed up with another provider type (PCP, endocrinologist, or rheumatologist).

Nonattendance for osteoporosis evaluation following a fragility fracture was associated with increased age (Table 1) and having private health insurance, whereas having government health insurance was associated with increased attendance (Table 2). Zip code distance from the BHC and neighborhood socioeconomic disadvantage were not significantly associated with post-fracture osteoporosis evaluation attendance.

The fragility fracture cases referred to the BHC included fractures of the distal radius, hip, proximal humerus, pelvis, and spine, as well as other fractures including toe, femur stress, clavicle, ankle, metacarpal, elbow, hand, femoral condyle, and patella fractures, which cumulatively made up less than 5% of fracture cases and were considered “other.” Type of fracture was significantly associated with attendance for osteoporosis evaluation (*p*=0.0147). Hip fracture was the predominant fragility fracture among those referred to the BHC and had the lowest attendance for osteoporosis evaluation following fracture (Table 3). Distal radius and other fracture types were the most likely to attend osteoporosis evaluation; compared to those with hip fracture, those with “other” fractures were 2.7 times more likely to attend evaluation and those with distal radius fracture were 1.7 times more likely to attend (Table 3). In unadjusted modified Poisson regression, hip fracture (RR: 0.66, CI: 0.48, 0.91) and age (RR: 0.88 per 10 years, CI: 0.81, 0.96) were each significantly associated with lower attendance for osteoporosis evaluation. In an adjusted model including both age and hip fracture, both remained independently significantly associated with lower attendance rates (Table 4). In addition, there was no difference in attendance for osteoporosis evaluation between patients that were hospitalized vs. not hospitalized for their fragility fracture (*p*=0.097).

## 4. Discussion

In this study, we sought to determine if patient demographic factors, insurance status, or fracture type were associated with nonattendance for osteoporosis evaluation after being referred to our BHC following a fragility fracture. Nonattendance was associated with older age and having private health insurance. Hip fracture was the most common fragility fracture referred to our BHC and was also associated with nonattendance. Patients with government health insurance were more likely to attend osteoporosis evaluation.

FLSs are considered one of the most effective strategies for the prevention of secondary fractures, but attendance is variable. Reports of attendance rates in the literature are limited and range from 20% to 86% [19]. In our study, the overall attendance rate for osteoporosis evaluation among patients referred to our BHC was 34.91%. In a previous questionnaire-based study assessing patient-related factors associated with attendance or nonattendance for osteoporosis evaluation, male gender, frailty, living alone, having low general education, or low interest in bone health and subsequent fracture risk were independently associated with nonattendance [23]. Similarly, we report that older patients were less likely to attend post-fracture osteoporosis evaluation. This may be due to associated frailty, other medical comorbidities, or logistical concerns which we did not explore in this study. In contrast, our study found no significant difference between male and female identifying patients in BHC attendance. In a clinical trial investigating the characteristics and motivations of nonparticipating patients when invited for osteoporosis screening, current alcohol use and smoking, age over 75 years, physical impairment, low BMI, and living alone were associated with nonattendance for a scheduled bone mineral density scan appointment [24]. The same study also reported no difference in education level, employment status, and personal income between attending and nonattending patients. In our study, ADI was used as a measure of socioeconomic disadvantage, and we did not find a difference in ADI between attending and nonattending patients. The majority of studies investigating FLS attendance were conducted in countries that utilize a universal healthcare system and thus did not evaluate health insurance type as a factor for FLS attendance. In the United States, the two main options for health insurance are private health insurance, provided through an employer or purchased from a health insurance company, or government health insurance, provided by the federal or state government. In addition, there are a variety of health insurance plans that patients can choose from. We examined the impact of insurance type in our United States–based study and found that patients with private health insurance were less likely to attend osteoporosis evaluation, whereas patients with government-based health insurance were more likely. Identification of risk factors for receiving inadequate post-fragility fracture care can inform strategies to reduce discrepancies in osteoporosis evaluation. For example, completing the formal osteoporosis evaluation for hip fracture patients prior to hospital discharge could begin the process while the patients are easily accessible and may impact rates of future osteoporosis treatment, when indicated.

In a study assessing attendance for a scheduled bone densitometry in patients at high risk for secondary fragility fractures, patients sustaining a hip fracture were less likely to attend, while patients with a wrist or ankle fracture were more likely to attend the visit [25]. Similarly, we found that hip fractures were both the most common type of fragility fracture referred for osteoporosis evaluation in our study and had the lowest attendance rate, although we noted that patients referred after proximal humerus, pelvis, and spine fragility fractures were also less likely to attend osteoporosis evaluation than those referred after wrist fracture. Among fragility fractures seen in men and women over the age of 50, hip fractures cause substantial morbidity and mortality [26]. The one-year mortality risk in patients aged 65 and over that sustained a hip fracture has been estimated to be 12%–37% and the 5-year mortality risk can even reach 60% in some elderly populations [27]. Of those that survive a hip fracture, half do not regain their pre-fracture functionality, and approximately 20% require some form of long-term care [28]. Furthermore, approximately half of hip fracture patients have a history of an additional fracture prior to their hip fracture [29]. A fragility fracture of the hip should serve as a warning sign and lead to osteoporosis evaluation and treatment initiation, if warranted, to decrease the risk of secondary fractures.

A treatment gap is apparent in patients at risk for osteoporosis [18, 30, 31]. A study assessing patterns of drug prescriptions before and after a fragility fracture event reported that only 19% of patients with hip fractures had been receiving treatment for osteoporosis before the fracture, and this percentage increased to only 21% after the fracture [32]. Certainly, there is a dire need to address the discrepancy between patients who warrant osteoporosis treatment and patients who actually receive it. Since patients at highest risk for secondary fractures, such as older patients or those who have sustained a hip fracture, may be frailer and have more difficulty attending post-fracture outpatient appointments, the best time to evaluate and treat for osteoporosis may be in the time window that they are inpatient and being treated for their fracture event. Indeed, low attendance for osteoporosis evaluation is but one barrier contributing to the osteoporosis care gap. Even if a patient is successfully screened and initiated on osteoporosis therapy, their prognosis is also dependent on adherence to the treatment regimen and routine follow-up. Further understanding of all barriers contributing to the care gap is needed to develop targeted strategies to ensure patients most at risk are screened and optimally treated for osteoporosis.

Our study does have limitations. First, because our study population was from a convenience sample of patients referred to our BHC from a single hospital, results may not be generalizable. Second, study data were collected through retrospective chart review of medical records available only within our institution, as we did not have access to medical records from outside of our institution. Therefore, it may be possible that the patients who did not attend osteoporosis evaluation within our system may have had osteoporosis evaluation with an outside clinician without our knowledge.

In conclusion, FLS programs have great potential to close the osteoporosis care gap, but they can only do so if patients attend for evaluation and treatment. Barriers for low FLS attendance continue to be investigated. Patients who may have more difficulty attending post-fracture appointments, such as older patients or those who sustained a hip fracture, may benefit from inpatient osteoporosis evaluation and treatment initiation at the time of their fracture.

## Figures and Tables

**Table 1:** Demographics of patients referred to the BHC.

	Nonattendance *n* = 330 (65%)	Attendance *n* = 177 (35%)	*p* value
Age, years (SD)	75.76 (2.1)	72.59 (2.08)	**0.0075**
Gender, %			0.5708
Female	77.58	80.23	
Male	22.42	19.77	
Race, %			0.9108
White	84.55	81.92	
Black	4.24	6.21	
Asian	1.52	1.69	
Hispanic	2.73	3.39	
Other	3.03	3.39	
Unknown	3.94	3.39	
English primary language, %	89.09	88.70	0.8829

*Note:p* value < 0.05 is considered significant. Bold values indicate statistical significance at *p* < 0.05.

Abbreviation: SD, standard deviation.

**Table 2:** Characteristics of patients referred to the BHC.

	Nonattendance *n* = 330 (65%)	Attendance *n* = 177 (35%)	*p* value
Zip code distance, mean miles (SD)	19.26 (67.84)	37.41 (190.70)	0.1204
ADI national, mean percentile (SD)	17.10 (12.93)	15.98 (11.77)	0.4283
ADI state, mean decile (SD)	3.939 (2.093)	3.802 (2.073)	0.5453
Insurance, %			**0.0403**
Private	33.84	24.86	
Government	67.73	74.01	
MVA/worker compensation	0.91	0.56	
Self-pay	2.73	0.56	

*Note:* Government health insurance includes Medicaid, Medicare, and MassHealth; *p* value < 0.05 is considered significant. Bold values indicate statistical significance at *p* < 0.05.

Abbreviations: ADI, area deprivation index; MVA, motor vehicle accident; SD, standard deviation.

**Table 3:** Fractures referred to the BHC.

Fracture type	Nonattendance *n* = 330 (65%)	Attendance *n* = 177 (35%)	Relative risk (95% CI)
Hip (*n* = 127)	95 (75%)	32 (25%)	Reference
Distal radius (*n* = 109)	62 (57%)	47 (43%)	1.7 (1.2, 2.5)
Proximal humerus (*n* = 91)	61 (67%)	30 (33%)	1.3 (0.9, 2.0)
aMulti (*n* = 47)	29 (62%)	18 (38%)	1.5 (0.9, 2.4)
bOther (*n* = 14)	4 (31%)	9 (69%)	2.7 (1.7, 4.4)
Pelvis (*n* = 41)	28 (68%)	13 (32%)	1.3 (0.7, 2.2)
Spine (78)	50 (64%)	28 (36%)	1.4 (0.9, 2.2)

^a^Multiple fractures refer specifically to fractures that occurred concurrently.

^b^Other fractures included toe, femur stress fx, clavicle, ankle, metacarpal, elbow, hand, femoral condyle, patella, and peri-prosthetic.

**Table 4:** Modified Poisson regression of age and hip fracture with osteoporosis evaluation attendance.

	Measure of association: RR (95% CI)	*p* value
*Unadjusted model*		
Age	0.88 (0.81, 0.96)	**0.0042**
Hip fracture	0.66 (0.48, 0.91)	**0.0120**

*Adjusted model*		
Age	0.90 (0.83, 0.98)	**0.0181**
Hip fracture	0.70 (0.50, 0.98)	**0.0359**

*Note:* Age RR is per 10 years; *p* value < 0.05 is considered significant. Bold values indicate statistical significance at *p* < 0.05.

Abbreviations: RR, relative risk; 95% CI, 95% confidence interval.

## Data Availability

The data used to support the findings of this study are restricted by the institution of the authors in order to protect patient privacy. Data are only available from the corresponding author for researchers who meet the criteria to access the confidential data.
